# Indoor and Outdoor Rodent Hosts of *Orientia tsutsugamushi*, Shandong Province, China

**DOI:** 10.3201/eid2710.210393

**Published:** 2021-10

**Authors:** Fei Li, Zhen-Tang Zhang, Li-Zhu Fang, Hao Yu, Xiang-Rong Qin, Xue-Jie Yu

**Affiliations:** Shandong University, Jinan, China (F. Li);; Huangdao District Center for Disease Control and Prevention, Qingdao, China (Z.-T. Zhang);; Wuhan University, Wuhan, China (L.-Z. Fang, H. Yu, X.-J. Yu);; The Second Hospital of Shandong University, Jinan (X.-R. Qin)

**Keywords:** *Orientia tsutsugamushi*, scrub typhus, rodents, animal host, Shandong, China, zoonoses, zoonotic infections, vector-borne diseases, mites, chiggers, bacteria, bacterial zoonoses

## Abstract

During December 2012–July 2016, we tested small indoor and outdoor mammals in Qingdao, China, for *Orientia tsutsugamushi* infection. We found that outdoor *Apodemus agrarius* mice, *Cricetulus barabensis* hamsters, and *Niviventer confucianus* rats, as well as indoor *Mus musculus* mice, tested positive for *O. tsutsugamushi* by PCR.

Scrub typhus is an emerging infectious disease caused by *Orientia tsutsugamushi* (*1*), which is transmitted through the bites of infected chiggers, the larvae of trombiculid mites of the genus *Leptotrombidium*. Scrub typhus has been documented in southern China for thousands of years (*2*) and emerged in northern China during the 1990s (*3*). Several studies have investigated the animal hosts of *O. tsutsugamushi* (*4*,*5*), but the major hosts and seasonality of *O. tsutsugamushi* in northern China remain unclear. We collected small animals in Qingdao, a city in eastern China, to investigate the hosts and seasonality of *O. tsutsugamushi*.

During December 2012–July 2016, we used indoor and outdoor mousetraps to capture 162 small mammals (154 rodents and 8 shrews) in 2 villages in Huangdao District, Qingdao (119°30′–121°00′E, 35°35′–37°09′N) (Figure). All animal samples were obtained in accordance with the Implementation Regulations of the People’s Republic of China on the Protection of Terrestrial Wild Animals (http://www.gov.cn/zhengce/2020-12/25/content_5574749.htm). The collection of rodents for microbiological studies was approved by the Ethics Committee of Prevention Medicine of Shandong University (Jinan, China; approval no. 20150501). 

**Figure:**
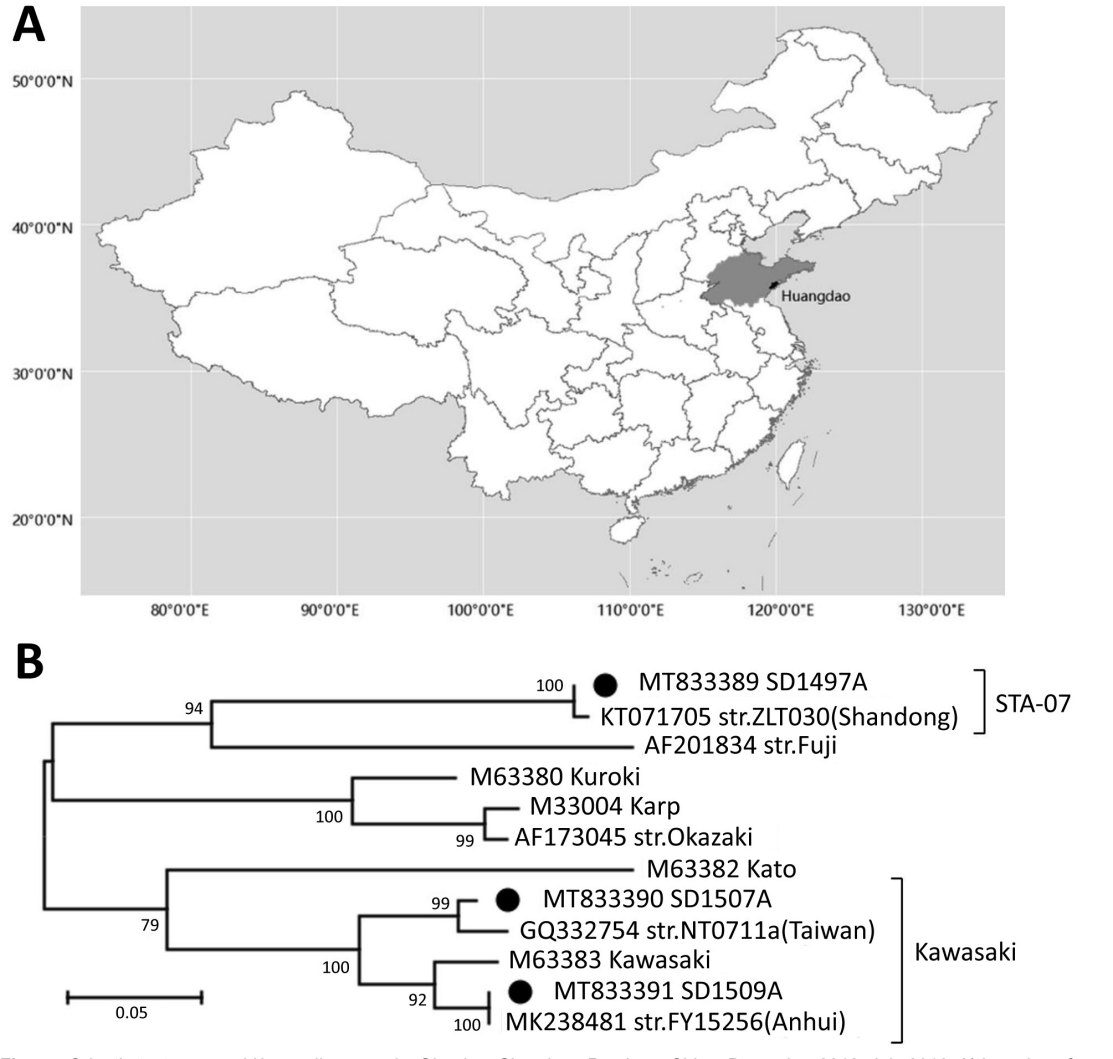
*Orientia tsutsugamushi* in small mammals, Qingdao, Shandong Province, China, December 2012–July 2016. A) Location of Shandong Province in China. B) Maximum-likelihood phylogenetic tree of *O. tsutsugamushi* constructed by MEGA version 7.0 (http://www.megasoftware.net). Black circles indicate strains isolated in this study. Numbers to the left of nodes indicate bootstrap values based on 1,000 replicates. Scale bar indicates number of nucleotide substitutions per site.

We classified samples by morphologic characteristics. We captured all 7 *Cricetulus barabensis* hamsters, 18 *Tscherskia triton* hamsters, and 8 *Niviventer confucianus* rats in the fields, as well as 98.6% (69/70) of *Apodemus agrarius* mice. We captured most *Rattus norvegicus* rats (22/24; 92%) and *Mus musculus* mice (18/27; 67%) in indoor settings, as well as 25% (2/8) of *Crocidura lasiura* shrews.

We extracted and screened DNA from rodent spleens for *O. tsutsugamushi* by nested PCR selective for the 56-kDa type-specific antigen gene with outer primers (5′- TCAAGCTTATTGCTAGTGCAATGTCTGC-3′ and 5′-AGGGATCCCTGCTGCTGTGCTTGCTGCG-3′) and inner primers (5′-GATCAAGCTTCCTCAGCCTACTATAATGCC-3′ and 5′-CTAGGGATCCCGACAGATGCACTATTAGGC-3′) (*6*,*7*). Overall, 4.5% of 154 rodents but none of the 8 shrews were positive for *O. tsutsugamushi*. 

All infected rodents were captured during autumn (i.e., September–November); among rodents captured in autumn, the infection rate was 8.1% (7/86; p>0.05 by 1-sided Fisher exact test). None of the 68 rodents captured during spring, summer, and winter tested positive for *O. tsutsugamushi* (Table). The absence of *O. tsutsugamushi* infection among rodents collected during spring, summer, and winter indicated that these rodents were not reservoirs but temporary amplifying hosts for *O. tsutsugamushi*. The presence of *O. tsutsugamushi* among rodents during autumn months is consistent with the seasonality of scrub typhus among patients in Shandong Province. Among humans, *O. tsutsugamushi* infections occur during September–December and peak in October (*8*), in alignment with the reproductive season of *Leptotrombidium scutellare* mites (*3*). 

**Table:** Prevalence of *Orientia tsutsugamushi* in small mammals, Qingdao, Shandong Province, China, December 2012–July 2016

Species	Season, no. positive/no. tested (%)				
	Spring	Summer	Autumn	Winter	Total
Rodent					
* Apodemus agrarius*	0/2 (0)	0/28 (0)	3/39 (7.7)	0/1 (0)	3/70 (4.3)
* Mus musculus*	0/2 (0)	0/14 (0)	2/9 (22.2)	0/2 (0)	2/27 (7.4)
* Rattus norvegicus*	0/4 (0)	0/8 (0)	0/9 (0)	0/3 (0)	0/24 (0)
* Tscherskia triton*	0/1 (0)	0/1 (0)	0/15 (0)	0/1 (0)	0/18 (0)
* Niviventer confucianus*	0	0	1/8 (12.5)	0	1/8 (12.5)
* Cricetulus barabensis*	0/1 (0)	0	1/6 (16.7)	0	1/7 (14.3)
Shrew					
* Crocidura lasiura*	0	0	0/8 (0)	0	0/8 (0)
Total	0/10 (0)	0/51 (0)	7/94 (7.4)	0/7 (0)	7/162 (4.3)

Overall, we found that 4.3% of *A. agrarius* mice, 7.4% of *M. musculus* mice, 12.5% of *N. confucianus* rats, and 14.3% of *C. barabensis* hamsters tested positive for *O. tsutsugamushi*; these results indicate the potential role of these rodents as animal hosts for *O. tsutsugamushi*. None of the 24 *R. norvegicus* rats, 18 *T. triton* hamsters, or 8 *C. lasiura* shrews tested positive, suggesting that these animals are not major hosts for *O. tsutsugamushi*. *R. norvegicus* rats, which were mainly captured indoors, were all negative for *O. tsutsugamushi*; these findings suggest that these rats might primarily stay indoors, thereby avoiding exposure to chiggers in the fields. We found 2 (11.1%) *O. tsutsugamushi*–positive indoor house mice, possibly reflecting their travels between house and field.

The sequences of *O. tsutsugamushi* from rodents identified belonged to 2 lineages, Kawasaki and STA-07 (Figure). We identified the Kawasaki strain in 4 rodent species collected in the same village during the autumns of 2013 and 2014 and the STA-07 strain in *A. agrarius* mice in a village 20 km away during the autumn of 2014. These results suggest that *O. tsutsugamushi* isolates from same geographic area are highly homologous regardless of host species. We deposited the 56-kDa type-specific antigen gene sequences obtained in this study in GenBank (accession nos. MT833389–95).

In conclusion, we documented *O. tsutsugamushi* infection among outdoor *A. agrarius* mice, *N. confucianus* rats, and *C. barabensis* hamsters, as well as indoor *M. musculus* mice, in Shandong Province; these rodents might serve as animal hosts for *O. tsutsugamushi*. The finding of *O. tsutsugamushi* infection among indoor mice suggest that persons might be exposed to chiggers and *O. tsutsugamushi* at home. Further study is needed to investigate whether scrub typhus patients in the area had a history of working or traveling in the fields and whether their houses were infested with mice and chiggers. Our results indicate that physicians should be attentive to patients who might have *O. tsutsugamushi* infection, even if those patients have not worked in the field.

## References

[1] Walker DH. *Rickettsiae*. In: Baron S, editor. Medical Microbiology. 4th ed. Galveston (TX): University of Texas Medical Branch at Galveston; 1996. 21413252

[2] Fan MY, Walker DH, Yu SR, Liu QH. Epidemiology and ecology of rickettsial diseases in the People’s Republic of China. Rev Infect Dis. 1987;9:823–40. 10.1093/clinids/9.4.823 3326129

[3] Zhang S, Song H, Liu Y, Li Q, Wang Y, Wu J, et al. Scrub typhus in previously unrecognized areas of endemicity in China. J Clin Microbiol. 2010;48:1241–4. 10.1128/JCM.01784-09 20129967 PMC2849583

[4] Liu YX, Jia N, Xing YB, Suo JJ, Du MM, Jia N, et al. Consistency of the key genotypes of *Orientia tsutsugamushi* in scrub typhus patients, rodents, and chiggers from a new endemic focus of northern China. Cell Biochem Biophys. 2013;67:1461–6. 10.1007/s12013-013-9646-0 23760611

[5] Wu G-H, Wang C-J, Li B-J, Jiang Z-K, Ding L-Y, Wang L. General situation on studies of animal hosts of tsutsugamushi disease in China. Chinese Journal of Hygienic Insecticides of Equipments. 2013;19:370–3.

[6] Tamura A, Yamamoto N, Koyama S, Makisaka Y, Takahashi M, Urabe K, et al. Epidemiological survey of *Orientia tsutsugamushi* distribution in field rodents in Saitama Prefecture, Japan, and discovery of a new type. Microbiol Immunol. 2001;45:439–46. 10.1111/j.1348-0421.2001.tb02643.x 11497219

[7] Furuya Y, Yoshida Y, Katayama T, Yamamoto S, Kawamura A Jr. Serotype-specific amplification of *Rickettsia tsutsugamushi* DNA by nested polymerase chain reaction. J Clin Microbiol. 1993;31:1637–40. 10.1128/jcm.31.6.1637-1640.1993 8315007 PMC265595

[8] Wu GH. The epidemiological characteristics and prevention and cure of scrub typhus in China. China Public Health. 2000;16:777–9.

